# Endothelial dysfunction due to the inhibition of the synthesis of nitric oxide: Proposal and characterization of an *in vitro* cellular model

**DOI:** 10.3389/fphys.2022.978378

**Published:** 2022-11-17

**Authors:** Fernanda Cardoso da Silva, Bruna Juber de Araújo, Carina Santos Cordeiro, Vinícius Marques Arruda, Bruno Quintanilha Faria, Joyce Ferreira Da Costa Guerra, Thaise Gonçalves De Araújo, Cristina Ribas Fürstenau

**Affiliations:** ^1^ Animal Cell Culture Laboratory, Institute of Biotechnology, Federal University of Uberlândia, Patos de Minas, MG, Brazil; ^2^ Laboratory of Vascular Biochemistry, Center for Natural and Human Sciences (CCNH), Federal University of ABC (UFABC), Santo André, SP, Brazil

**Keywords:** endothelial dysfunction, nitric oxide, p22^phox^, NOX4, IL-6, ACE, *in vitro* model

## Abstract

The vascular endothelium plays a pivotal role in the maintenance of vascular homeostasis, mediated by vasoactive molecules produced by endothelial cells. The balance between vasoconstrictor and vasodilator biomolecules is what guarantees this equilibrium. Therefore, an increase in the bioavailability of vasoconstrictors along with a reduction in vasodilators may indicate a condition known as endothelial dysfunction. Endothelial dysfunction is marked by an inflammatory process and reduced activity of vasoprotective enzymes, being characterized by some factors like the reduction of the bioavailability of nitric oxide (NO) and increase in the production of reactive oxygen species (ROS), pro-inflammatory and vasoconstrictor molecules. This condition is a predictive marker of several cardiovascular diseases (e.g., atherosclerosis, hypertension, and diabetes). Research is affected by the scarcity of suitable *in vitro* models that simulate endothelial dysfunction. The goal of this study was to induce an *in vitro* condition to mimic endothelial dysfunction by inhibiting NO synthesis in cells. Thymus-derived endothelial cells (tEnd.1) were treated with different concentrations of L-NAME (from 1 to 1,000 μM) for different times (12, 24, 48, 72, 96, and 120 h without and with retreatment every 24 h). Cell viability, nitrite concentration, p22^phox^, NOX2, NOX4, IL-6, and ACE genes expression and lipid peroxidation were evaluated. The results indicate that the treatment with 100 μM L-NAME for 72 h without retreatment reduced NO concentration and NOX4 gene expression while increasing ACE expression, thus mimicking reduced vascular protection and possibly increased vasoconstriction. On the other hand, treatment with 100 μM L-NAME for 96 h with retreatment reduced the concentration of NO and the expression of the p22^phox^ gene while increasing the expression of the IL-6 and ACE genes, mimicking the increase in inflammation and vasoconstriction parameters. Based on these results, we thus propose that both 100 μM L-NAME for 72 h without retreatment and 100 μM L-NAME for 96 h with retreatment may be used as models for *in vitro* endothelial dysfunction according to the purpose of the study to be conducted.

## Introduction

The endothelium consists of a cell monolayer and forms the inner lining of blood vessels (Krüger-Genge et al., 2019). These cells not only act as a tissue barrier because of tight junctions (Cong and Kong, 2020), but also exhibit important biological functions, producing very active biomolecules in the vasculature (Sena et al., 2013) that are essential for the integrity and maintenance of vascular homeostasis (Krüger-Genge et al., 2019). Also, they perform important functions as regulators of angiogenesis, regeneration, cell differentiation (Ribatti et al., 2021), vascular tone, blood flow (Krüger-Genge et al., 2019), immune responses (Sturtzel, 2017) and cellular permeability, influencing in capillary transport (Daiber et al., 2019). This diversity of functions reflects the heterogeneity of these cells along the vascular tree (Hennigs et al., 2021).

In order to perform such diverse functions, endothelial cells produce a range of important biomolecules, such as endothelium-derived hyperpolarizing factor (EDHF), nitric oxide (NO) and prostacyclin (PGI2), which present vasodilator and antiproliferative effects on vascular smooth muscle cells (Garland and Dora, 2021). On the other hand, endothelial cells also generate endothelin-1 (ET-1), angiotensin II (AT-II) and reactive oxygen species (ROS), which present vasoconstrictor effects and promote the proliferation of vascular smooth muscle cells (Sena et al., 2013).

Specifically, we highlight the production of NO and ROS in endothelial cells. NO is synthesized by three subtypes of the NO synthase enzyme (NOS): neuronal NOS (nNOS, NOS1), inducible NOS (iNOS, NOS2) and endothelial NOS (eNOS, NOS3). The most expressed in endothelial cells is eNOS. These enzymes need L-arginine and O2 to catalyze the reaction that leads to the production of NO and l-citrulline (Cyr et al., 2020). All NOS isoforms are active in the form of dimers (Yuyun et al., 2018) and require cofactors and coenzymes for their activation, such as O_2_, NADPH, flavins and biopterins (Flora Filho and Zilberstein, 2000).

ROS are produced by different sources within the cell. Among the reactive species, we highlight the superoxide anion (O2º^−^), hydrogen peroxide (H_2_O_2_), hydroxyl radical (OH^−^) and peroxynitrite (ONOO^−^). The sources are the mitochondrial electron transport chain NADPH oxidase (NOX), uncoupled eNOS and xanthine oxidase (Incalza et al., 2018). Here, we highlight NOX, which are the only enzymes whose primary function is the generation of ROS. They are an enzymatic complex consisting of five main components, two of which are membrane-associated, gp91^phox^ and p22^phox^, and three cytosolic, p47^phox^, p67^phox^ and Rac1 or Rac2 (Babior, 2000). NOX mainly generate H_2_O_2_ and O2º^−^ (Drummond and Sobey, 2014), and in endothelial cells NOX1, NOX2 and NOX5 generate O2º^−^, and NOX4 generate H_2_O_2_ (Drummond and Sobey, 2014; Langbein et al., 2016).

Endothelial dysfunction (ED) is a condition characterized by an inflammatory process concomitant with oxidative stress, which causes loss of endothelial function and consequent imbalance in the production of biomolecules (Daiber et al., 2019). Two of the most important events that characterize ED are the reduction in NO bioavailability and a considerable increase in ROS production, such as superoxide anion (Rudic and Sessa, 1999; Vanhoutte et al., 2017; Incalza et al., 2018). In addition, the production of protective vascular biomolecules is reduced and there is an increase in leukocyte adhesion and permeability and in endothelial cell senescence (Xu et al., 2021). This condition stands out as a pathological mechanism that is related to a variety of factors and is pointed as a predictor of distinct cardiovascular, metabolic and inflammatory diseases (Daiber et al., 2019; Vincent et al., 2021).

Thus, it is clear that endothelial dysfunction is a conserved target in metabolic disorders. Considering that different metabolic and cardiovascular diseases may coexist in the same patient and that ED is a common event in these diseases, endothelial dysfunction may also be used as a target for new therapies (Jamwal and Sharma, 2018; Xu et al., 2021). It is therefore necessary to deepen the studies regarding ED to better understand the mechanisms and pathways involved in this condition.

L-NAME is a prodrug with an inhibitory capacity for NO synthesis (Pfeiffer et al., 1996), as it is an analogue of L-arginine. The inhibition of NOS by L-NAME occurs through the binding of this inhibitor to the enzyme at the catalytic site, competing with the substrate and preventing the binding of L-arginine (Rees et al., 1990; Peterson et al., 1992). In addition, L-NAME is a reversible and non-selective inhibitor of NOSs commonly used in long- and short-term experiments, either *in vitro* or *in vivo*, with the aim of identifying events associated with the restriction of NO production (Kopincová et al., 2012).

Knowing that ED is common in patients with essential hypertension and that it is closely related to reduce NO bioavailability, an animal model of hypertension induction was previously developed using L-NAME (Rees et al., 1990) (REES, et al., 1990) and is now widely used. To date, however, a model of induction of an ED-like condition in cell culture that similarly follows this model used *in vivo* has not yet been described. This study aimed to develop a protocol to simulate endothelial dysfunction *in vitro* by inhibiting NO synthesis in thymus-derived endothelial cells (tEnd.1).

## Materials and methods

### Cell culture

The murine thymic endothelioma cell line (tEnd.1) was established by means of transformation with the polyomavirus medium T oncogene. It has been proven to maintain the functional properties of normal endothelium (Williams et al., 1988; Boraschi et al., 1991), in addition to the fact that it is already known to have a high NOS activity, expression of the endothelial-NOS isoforms (eNOS) and inducible-NOS (iNOS) (Arese et al., 1995). Another factor also considered in this work is that this strain presents a greater activity of NO synthesis than the untransformed strains. Maximal NO synthase (NOS) activity was about 200-fold higher in cell lysates from the endothelioma cell line tEnd.1 than in lysates from untransformed controls (Ghigo et al., 1995). In this sense, the use of this strain allows a better analysis of the inhibition of NO production by L-NAME.

Murine thymus-derived endothelial cell line tEnd.1 (RRID: CVCL_62 72) was cultured in Dulbecco’s modified Eagle’s medium (DMEM, GIBCO^®^), enriched with 10% fetal bovine serum (FBS, GIBCO^®^), 100 U/ml penicillin and 100 μg/ml streptomycin (GIBCO^®^) at 37°C, 5% CO_2_ in an humidified incubator until reaching 80%–90% confluence. Cells were used between the third and eighth passages. Depending on the experiment, cells were plated in 6, 12 or 96-well plates, with 2 × 10^5^ cells/well, 4 × 10^4^ cells/well and 1 × 10^3^ cells/well, respectively.

### L-NAME treatment

There was a waiting period of 24 h after plating for the cells to adhere and only then was the treatment started. Cells were made quiescent by fetal bovine serum deprivation (0.5%) for 3 h and subsequently subjected to L-NAME (SIGMA^®^) treatment (1, 10, 100, and 1 mM) or 100 μM L-arginine (SIGMA^®^) treatment as a negative control (Klawitter et al., 2017) for 12, 24, 48, 72, 96, and 120 h with and without retreatment every 24 h. Retreatments were performed every 24 h by replacing the “old culture medium” with the “fresh culture medium” with the same treatment. For all analyses, a sample n of five wells from three different cell cultures was used.

### Cell viability

Following cell treatments, 25 μl of Tetrazolium Blue Thiazolyl Bromide (MTT) (Ludwig Biotec^®^) was added at a concentration of 5 mg/ml in PBS (w/v) to each well and plates were left for 4 h in the incubator. The culture medium with MTT excess was aspirated, followed by the addition of dimethyl sulfoxide (DMSO) to dissolve formazan crystals (Mosmann, 1983). The MTT method is based on the ability of living cells to reduce the yellow tetrazolium salt to the purple insoluble formazan, which precipitates due to the action of the mitochondrial enzyme succinyl dehydrogenase, active only on living cells (Mosmann, 1983). Optical reading was performed on an automatic plate reader at 560 nm (Readwell PLATE, ROBONIK^®)^. Cell viability results were obtained according to Eq. 1:

$$\%\;Cell\;viability=\frac{\left(A_{t}-A_{b\;}\right)}{\left(A_{c}-A_{b\;}\right)}\times 100$$ (1)

where:*A*_*t*_: Absorbance at 560 nm of “treated” cells (cells + treatment).*A*_*b*_: Absorbance at 560 nm of “blank” wells (only DMSO).*A*_*c*_: Absorbance at 560 nm of “control” cells [culture medium only (no treatment) + cells].

### Nitrite quantification

Nitrite quantification was performed as an indirect measurement of NO levels. The treatments were performed as described before, but using phenol red free DMEM (GIBCO^®^, Grand Island, New York, United States) not to influence the readings. Nitrite content was determined using a Griess reagent kit (Thermo Fisher Scientific^®^) according to the manufacturer’s instructions. The culture medium from each well was collected, centrifuged at 16,000 rpm and 4°C (Hermle Labor Technik, Z 36 HK) for 10 min; the supernatant was kept for further analysis. In a 96-well microplate, 20 µl Griess reagent, 150 µl of nitrite-containing sample and 130 µl deionized water were mixed. After 30 min of incubation in the dark at room temperature, the plate was read on an ELISA plate reader (Readwell PLATE, ROBONIK^®^) at 560 nm. Nitrite concentration in the samples was calculated based on a standard curve of different sodium nitrite concentrations (1, 5, 10, 30, 50 and 100 µM).

### Real-time PCR gene expression analysis

After treatments, total cellular RNAs were extracted with Trizol^®^ reagent (Invitrogen, Carlsbad, CA, United States) following the manufacturer’s instructions. Reverse transcription was performed as previously described (Mota et al., 2019). The reference gene β-actin was used (5′GGG​AAA​TCG​TGC​GTG​ACA​TC3′ and 5′GCC​ACA​GGA​TTC​CAT​ACC​CAA3′) to validate RNA quality of each sample and for normalization of qPCR assays. For validation, conventional PCR reactions were performed as follows: 2.0 µl of cDNA amplicons, 1.0 U of Taq DNA Polymerase Platinum (Invitrogen), 50 mM KCl; 10 mM Tris–HCl pH 8.3, 2.0 mM MgCl_2_, 200 μM dNTPs and 5.0 pmol of each primer. All components were incubated for 25 cycles at 94°C 30 s, 60°C 1 min, 72°C 1 min, preceded by an initial denaturation at 95°C for 5 min.

For qPCR, each 2 µl aliquot of cDNA was amplified with 5 pmol of each specific primer (EXXTEND^®^) for eNOS (Srinivasan et al., 2004), p47^phox^ (5′ATC​CCC​AGC​CAG​CAC​TAT​GTG3′ and 5′GAG​ATC​CAC​ACA​AGA​GAA​CAG​AG3′), p22^phox^ (5′CCA​GTG​TGA​TCT​ATC​TGC​TGG​CA3′ and 5′GCC​TCC​TCT​TCA​CCC​TCA​CTC3′), NOX2 (Emmerson et al., 2018), IL-6 (5′TGC​TAC​CAA​ACT​GGA​TAT​AAT​CAG​GAA3′ and 5′CTC​TGA​ACT​CTG​GCT​TTG​TC3′), NOX4 (Dasgupta et al., 2020), and ACE (5′AGT​AGA​TTC​TGC​TCA​TGT​TGC​TT3′ and 5′GGC​CAA​GGA​GTT​GTA​GAT​GAG​TC3′). The reactions were conducted in six replicates in a total volume of 10 μl containing Power SYBR_ Green PCR Master Mix (Applied Biosystems—Carlsbad, CA, United States) in a thermal cycler (StepOnePlus™ Real-Time PCR System, Applied Biosystems). Standard relative curves for all primers were constructed and expression of each gene was quantified through comparative Cq method.

### Determination of lipid peroxidation

The quantification of lipid peroxidation is essential to evaluate oxidative stress in pathophysiological processes. One of the main products of lipid peroxidation is malondialdehyde (MDA), the most abundant aldehyde generated by the attack of reactive species on polyunsaturated fatty acids in cell membranes (Buege and Aust, 1978). MDA levels were determined by testing thiobarbituric acid reactive substances using the method of Buege and Aust (1978), which is based on the ability of the thiobarbituric acid (TBA) to bind to oxidized lipids. Briefly, 2 × 10^7^ cells submitted to different treatments were homogenized in 1 ml of cold 20 mM Tris HCL (pH 7.4) buffer. All the homogenate was mixed with trichloroacetic acid (28% w/v in 0.25 N HCl), TBA (1% in 0.25 M acetic acid) and BHT (125 mM in ethanol), heated for 1 h at 95°C and then placed in an ice bath. The precipitate was then removed by centrifugation at 10,000 × g for 15 min at 4°C, and the supernatant absorbance was determined at 535 nm in a spectrophotometer (Gehaka, UV-340G). MDA levels were calculated using 1,1,3,3-tetramethoxypropane as standard for constructing the calibration curve (12.5, 6.25, 3.125, 1.562, 0.781 and 0.390 µmoL/L).

### Statistical analysis

Results are presented as mean ± standard deviation for each of the measurements performed. Sample number (n) represents the number of experiments performed with different treatments in the cell line culture. For the comparison between groups, two-way analysis of variance (ANOVA) was applied, and Tukey’s and Dunnett’s multiple comparisons tests were used as *post-hoc* test because the distribution was normal. Differences between groups were considered significant at *p* < 0.05. Data were analyzed using GraphPad Prism software, version 7.00, for Windows.

## Results

### Cell viability

The MTT assay shows how treatments affected the viability of tEnd.1 cells. In groups without retreatment, 100 μM L-arginine increased cell viability (18.67%) after 48 h, while reducing this parameter (28.77% and 37.52%) after 96 and 120 h, respectively (Supplementary Figure S1). In general, L-NAME increased cell viability after 24, 48, 96 and 120 h of treatment. Differences were more evident in L-NAME-treated cells at any concentration for 24 h (increased cell viability of approximately 40%); 10 and 100 μM L-NAME for 48 h (increased cell viability of 33.85% and 34.83%, respectively); 100 µM for 96 h (increased cell viability of 22.88%); 10, 100 μM and 1 mM for 120 h (increased cell viability of 63.52%, 21.76% and 22.29%, respectively) (Supplementary Figure S1).

In groups that experienced retreatment every 24 h (Supplementary Figure S2), 100 μM L-arginine generally did not affect cell viability, except after 72 h with reduction of 26,04% in this parameter. Once again, L-NAME increased cell viability after 24, 48 and 72 h of treatment (Supplementary Figure S2). Comparing to untreated cells, after 24 h L-NAME, at any concentration, increased cell viability by approximately 40%. In the same way, 100 μM and 1 mM L-NAME after 48 h also increased cell viability around 38.22% and 27.51%, respectively.

**FIGURE 1:**
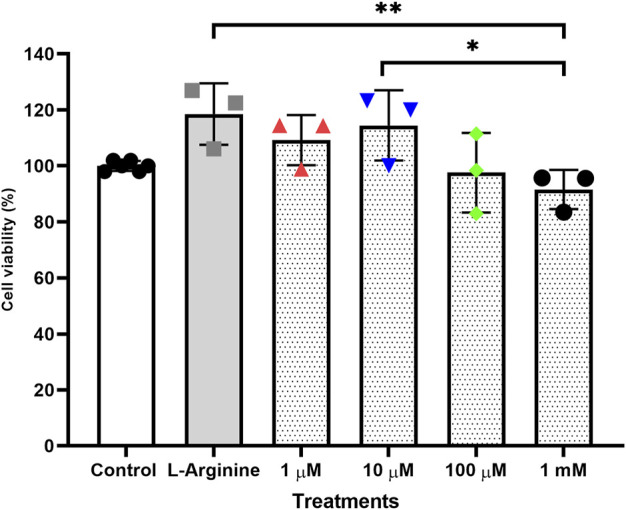
Cell viability of the thymus-derived endothelial cell line tEnd.1 after treatment with L-arginine (100 µM) and L-NAME (1, 10, 100 μM, 1 mM) for 72 h without retreatment. Data were compared by Two-way ANOVA, followed by Tukey’s test. (*Represents statistically significant difference between groups for *p* < 0.05; **Represents statistically significant difference between groups for *p* < 0.01).

**FIGURE 2:**
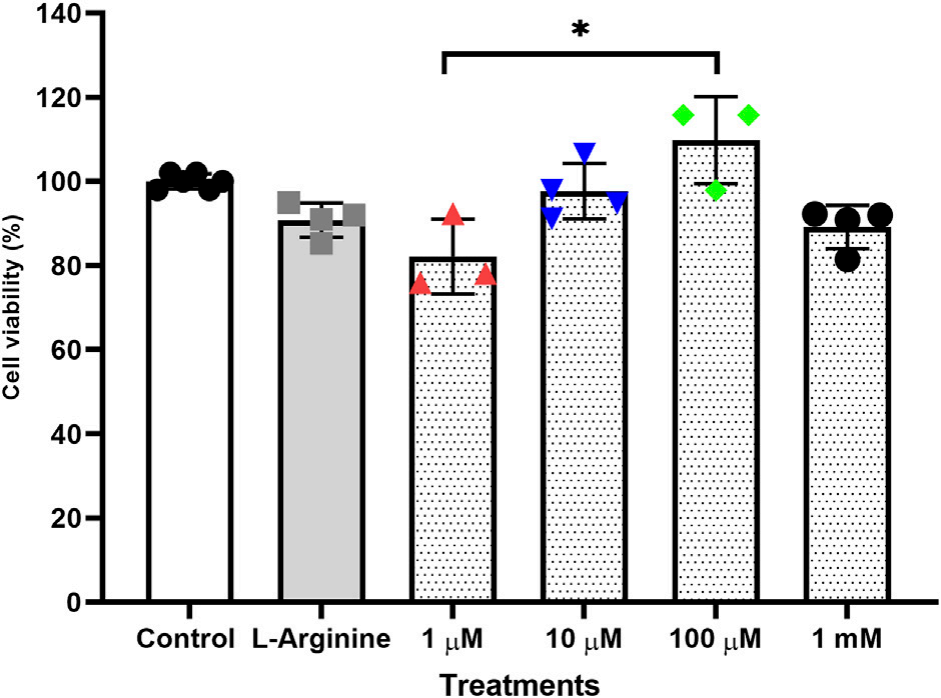
Cell viability of the thymus-derived endothelial cell line tEnd.1 after treatment with L-arginine (100 µM) and L-NAME (1, 10, 100 μM, 1 mM) for 96 h with retreatment. Data were compared by Two-way ANOVA, followed by Tukey’s test. (*Represents statistically significant difference between groups for *p* < 0.05; **Represents statistically significant difference between groups for *p* < 0.01).

Treatments performed for 72 h without retreatment (Figure 1) and treatments for 96 h with retreatment every 24 h (Figure 2) were the ones that least influenced cell viability when compared to the untreated cells, both treatments were established with the best conditions for carrying out gene expression analyses.

### Nitrite quantification

Results presented in Supplementary Figures S3, S4 show that in both groups (without and with retreatment) nitrite concentration increases in the control group until it reaches a peak at 72 h, and then decreases.

**FIGURE 3:**
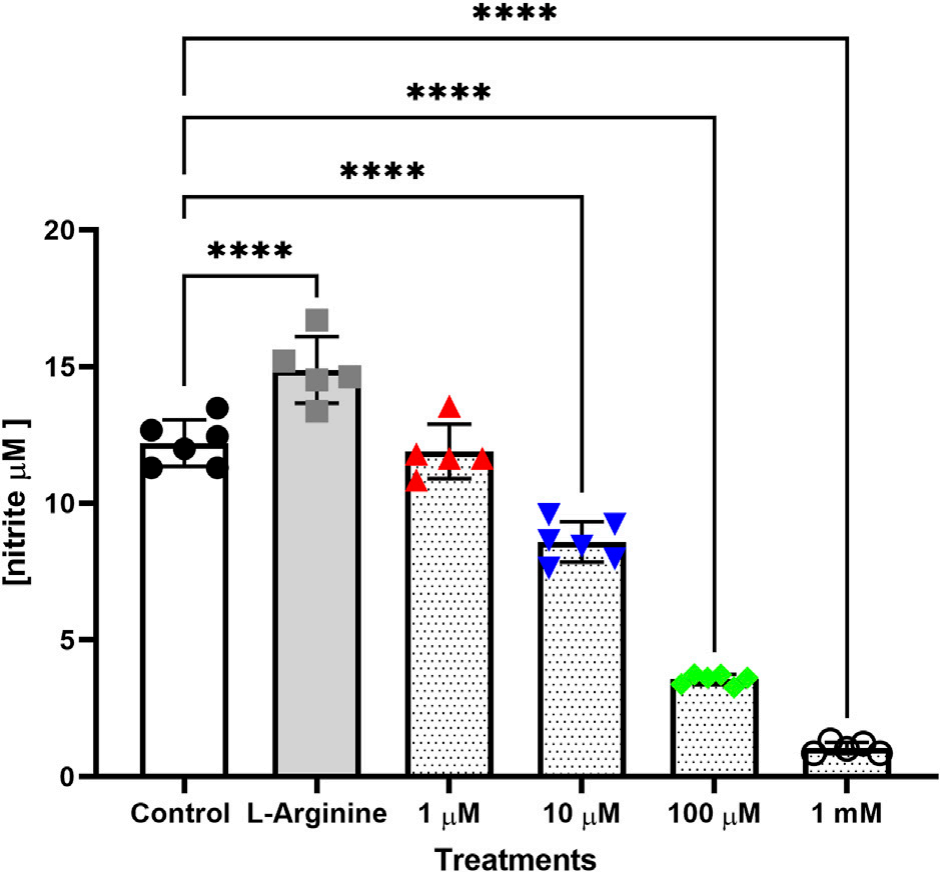
Nitrite concentration in culture medium of the thymus-derived endothelial cell line tEnd.1after treatment with L-arginine (100 µM) and L-NAME (1, 10, 100 μM, 1 mM) for 72 h without retreatment. Data were compared by Two-way ANOVA, followed by Tukey’s test. (****Represents significant difference between groups, for *p* < 0.0001).

Treatment with L-arginine increased nitrite concentration in the culture medium, which was more evident after 72, 96 and 120 h, with an increase of 21.98%, 61.90% and 62.68%, respectively when compared to untreated cells (Supplementary Figure S3). L-NAME at different concentrations reduced nitrite concentration in the culture medium in a dose-dependent manner. This reduction was more evident in 10, 100 μM and 1 mM L-NAME after 24, 48, 72, 96 and 120 h without retreatment. The largest reductions were observed with 1 mM L-NAME (91.37%), 100 μM L-NAME (70.83%) and 10 μM L-NAME (70.35%) after 72 h of treatment, when compared to untreated cells (Figure 3).

**FIGURE 4:**
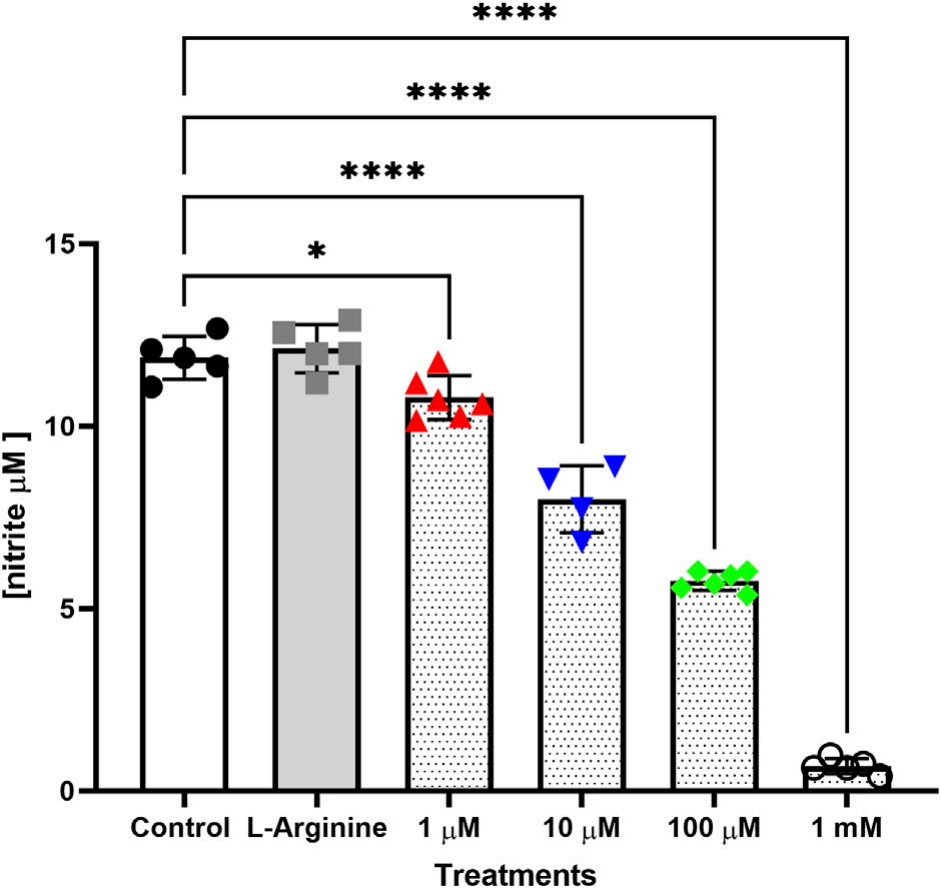
Nitrite concentration in culture medium of the thymus-derived endothelial cell line tEnd.1 after treatment with L-arginine (100 µM) and L-NAME (1, 10, 100 μM, 1 mM) for 96 h with retreatment every 24 h. Data were compared by Two-way ANOVA, followed by Tukey’s test (*Represents statistically significant difference between groups for *p* < 0.05; ****Represents significant difference between groups, for *p* < 0.0001).

In groups with retreatment (Supplementary Figure S4), L-NAME significantly reduced nitrite concentrations as follows: 62.05% at 100 μM and 92.60% at 1 mM after 48 h; 24.10% at 10 μM, 69.55% at 100 μM and 95.14% at 1 mM after 72 h; and 86.01% at 1 mM after 120 h. After 96 h with retreatment, results were more uniform and consistent with previous reports (Rees et al., 1990), with reductions of 32.62% at 10 μM, 51.47% at 100 μM and 94.30% at 1 mM L-NAME (Figure 4).

As endothelial dysfunction is characterized by reduced NO bioavailability, cells treated with 10 and 100 μM L-NAME for 72 h without retreatment and for 96 h with retreatment were chosen for subsequent experiments since they significantly reduced nitrite concentration and did not affect cell viability. Cells treated with 100 μM L-arginine were used as negative control at the same times of treatment.

### Real-time PCR gene expression analysis

**FIGURE 5:**
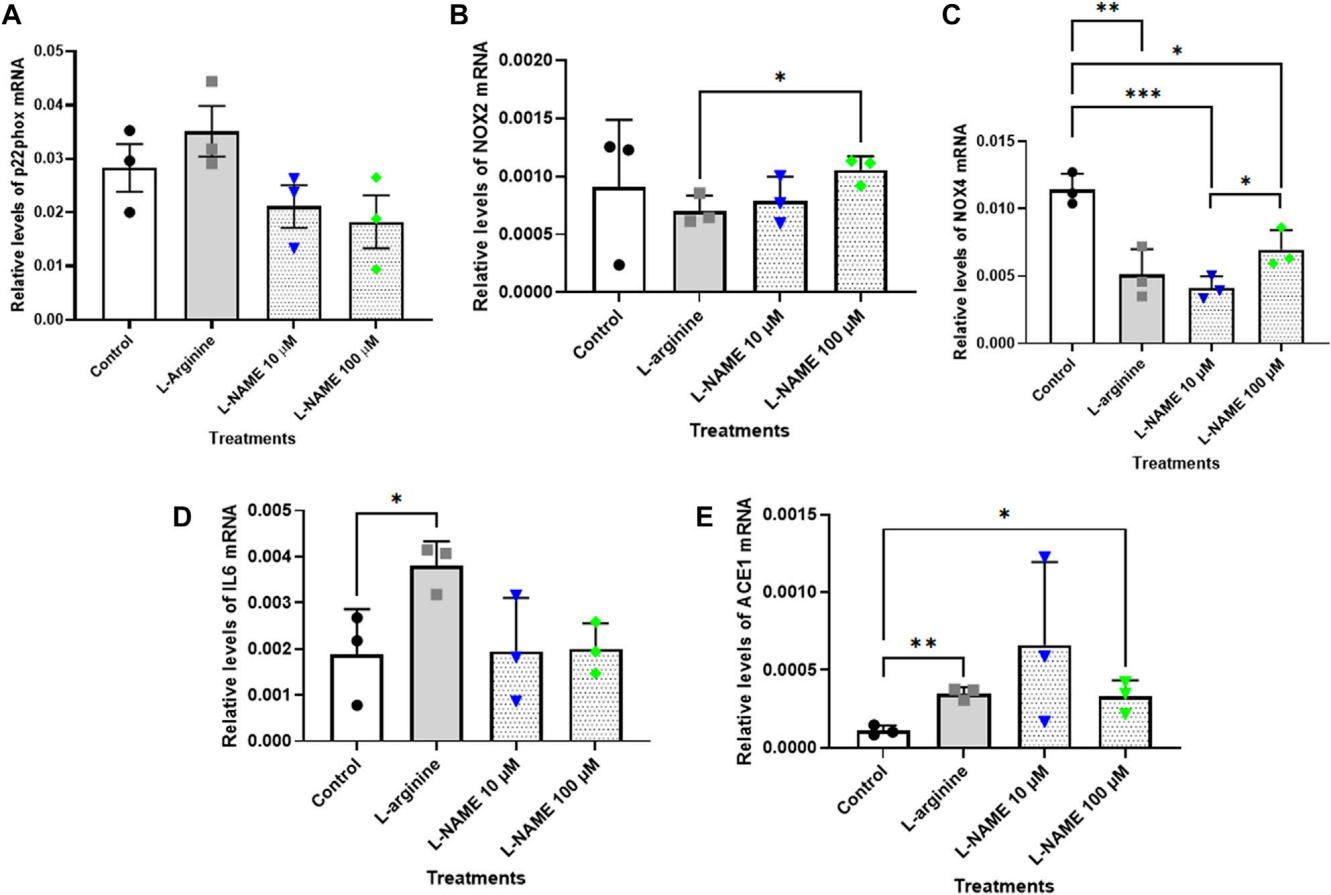
Relative levels of **(A)** p22^phox^, **(B)** NOX2, **(C)** NOX4, **(D)** IL-6 and **(E)** ACE mRNA in the thymus-derived endothelial cell line tEnd.1 after treatment with L-arginine (100 µM) and L-NAME (10 and 100 µM) for 72 h without retreatment. Data (mean, *n* = 3) were compared by T Tests. (*Represents statistically significant difference between groups for *p* < 0.05; **Represents statistically significant difference between groups for *p* < 0.01; ***Represents statistically significant difference from the control group for *p* < 0.001).

**FIGURE 6:**
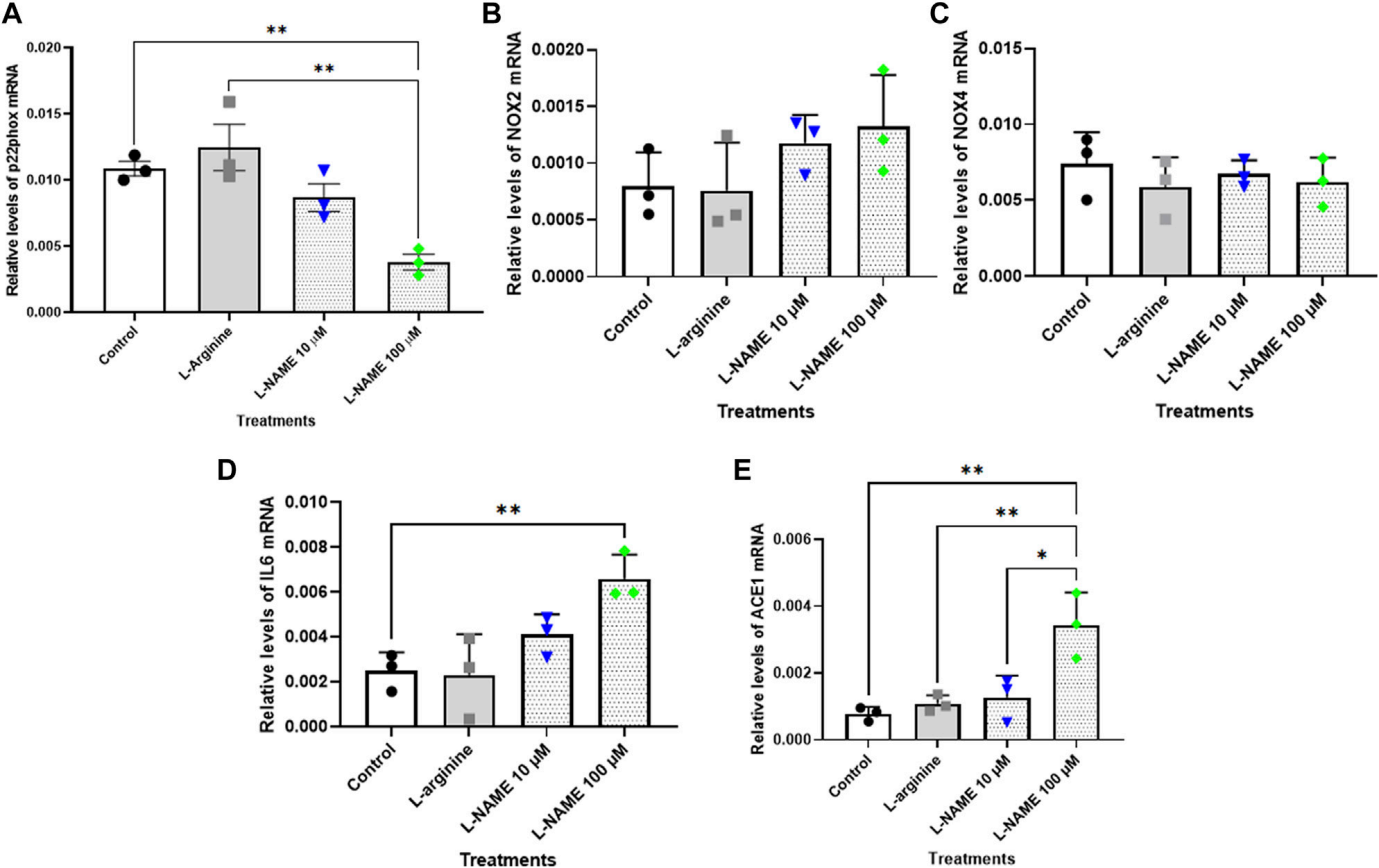
Relative levels of **(A)** p22^phox^, **(B)** NOX2, **(C)** NOX4, **(D)** IL-6 and **(E)** ACE mRNA in the thymus-derived endothelial cell line tEnd.1 after treatment with L-arginine (100 µM) and L-NAME (10 and 100 µM) for 96 h with retreatment. Data (mean, *n* = 3) were compared by T Tests. (*Represents statistically significant difference between groups for *p* < 0.05; **Represents statistically significant difference between groups for *p* < 0.01).

The results showed that relative levels of p22^phox^ mRNA did not differ after the treatments herein proposed for 72 h (Figure 5A). On the other hand, the treatment with 100 μM L-NAME for 96 h with retreatment exhibited a significant reduction of approximately 65% in p22^phox^ mRNA levels when compared with control group (Figure 6A). It is noteworthy that the expression of p22^phox^ gene in tEnd.1 cells was firstly reported in the present study. No amplification for the p47^phox^ and eNOS genes were obtained with the selected primers.

The results for NOX2 gene show that there was no statistically significant difference between the control and treated cells after 72 h without retreatment and after 96 h with retreatment (Figures 5B, 6B). Likewise, the NOX4 gene expression was not affected in cells treated with 10 and 100 μM L-NAME for 96 h with retreatment (Figure 6C). On the other hand, a reduction in NOX4 expression of approximately 55%, 64% and 40% was observed in cells treated with 100 μM L-arginine, 10 μM L-NAME and 100 μM L-NAME, respectively, per 72 h without retreatment (Figure 5C).

IL-6 mRNA expression was significantly increased in cells submitted to 100 μM L-arginine for 72 h without retreatment [approximately 100% increase when compared to the control (Figure 5D)]; and to treatment with 100 µM of L-NAME for 96 h with retreatment [approximately 160% increase when compared to untreated cells (Figure 6D)].

Besides, our results also showed that treatment with 100 μM L-arginine and 100 μM L-NAME for 72 h without retreatment increased ACE gene expression by 216% and 197%, respectively, when compared to untreated cells (Figure 5E). An increase in ACE expression of approximately 34% was also observed in t. End1 cells submitted to 100 μM L-NAME for 96 h with retreatment (Figure 6E).

### Determination of lipid peroxidation

**FIGURE 7:**
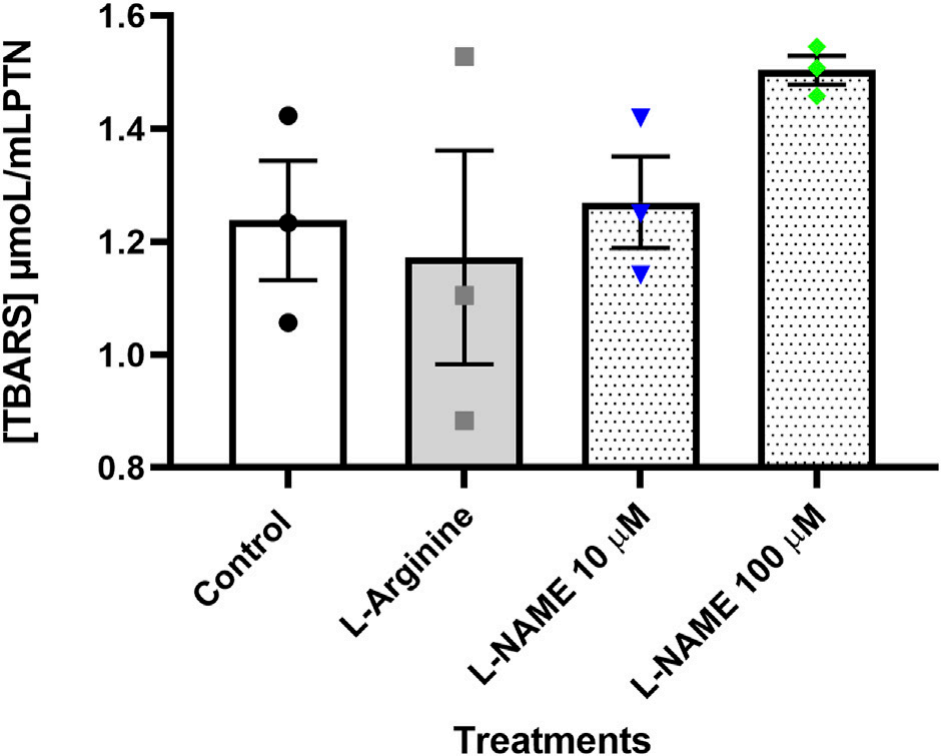
Concentration of thiobarbituric acid reactive substances (TBARS) given in µmoL/ml PTN in the thymus-derived endothelial cell line tEnd.1 medium after treatment with L-arginine (100 µM) and L-NAME (10 and 100 µM) for 72 h without retreatment. Data (mean, *n* = 3) were compared by Two-way ANOVA. No statistical differences were observed between groups.

Thiobarbituric acid reactive substances (TBARS) were quantified to check whether lipid peroxidation, a common event in endothelial dysfunction, was occurring in cells submitted to different treatments. The results obtained showed that there was no significant difference between the different treatments (Figure 7).

## Discussion

In the present study, we aim to propose a simple and accessible *in vitro* model for the study of endothelial dysfunction (ED) based on decreased NO bioavailability. Different concentrations of L-NAME at distinct time points (with and without cell retreatment) were tested to determine the best experimental conditions. In addition to the assessment of nitrite levels and cell viability, factors involved in the production of ROS, vasoconstrictors, and inflammatory agents, all characteristic of ED, were investigated by analyzing the gene expression of p22^phox^, NOX2, NOX4, ACE, and IL-6. The treatment with 100 μM L-NAME for 72 h without retreatment reduced NO concentration and NOX4 transcripts and increased ACE expression, mimicking reduced vascular protection and possibly increased vasoconstriction. Besides, treatment with 100 μM L-NAME for 96 h with retreatment reduced the concentration of NO and the expression of the p22^phox^ gene and increased IL-6 and ACE genes expression, mimicking the increase in inflammation and vasoconstriction parameters. Based on these results, we thus propose that both 100 μM L-NAME for 72 h without retreatment and 100 μM L-NAME for 96 h with retreatment may be used as *in vitro* models of ED according to the purpose of the study to be conducted.

ED is a primary condition of many cardiovascular diseases but is still little explored as a target for diagnosis and treatment (Mordi et al., 2016; Yang et al., 2016; Poredos and Jezovnik, 2018; Shaito et al., 2022). Therefore, interest in studying this condition has gradually grown over the years and studies focusing on the evaluation of endothelial function have shown to be very promising not only for diagnoses and therapies aimed at cardiovascular diseases, but also for other diseases that may be related, like COVID-19 (Todiras et al., 2017; AndriantoAl-Farabi et al., 2021).

Researchers have already proposed that certain treatments may induce a condition like endothelial dysfunction in cell culture when they mimic the metabolic changes inherent to this pathological state. It is well known that the treatment with native and oxidized low-density lipoproteins, angiostatin, homocysteine and high glucose rates can cause eNOS uncoupling, inducing a state similar to ED (Incalza et al., 2018). Other works induce endothelial dysfunction through the induction of endothelial cell autophagy or through the induction of oxidative stress (Gupta et al., 2022; Hua et al., 2022). However, from previous results of our research group in which L-NAME was used for the induction of secondary hypertension *in vivo* (Fürstenau et al., 2010), we found the need to develop an *in vitro* model to mimic this condition, especially ED, following a similar method of treatment to study other aspects related to the development and establishment of the disease. In this sense, one can observe that *in vitro* research is also of great importance for a better understanding of ED and to elucidate the pathways involved in endothelial cells. For these reasons, there is a crescent need for standardization of a method of inducing *in vitro* endothelial dysfunction (Aman et al., 2016; Gallogly et al., 2021; Jimenez Trinidad et al., 2021).

Initially, we evaluated how treatments affected endothelial cell viability. It is already known that NO affects the viability of endothelial cells and inhibits cell apoptosis induced by inflammation or atherosclerotic factors (Ziche et al., 1994; Dimmeler and Zeiher, 1999; Yuyun et al., 2018; Cyr et al., 2020). Therefore, in general, treatments that increase NO production are expected to increase cell proliferation, while those that reduce it led to cell death. L-arginine is an important amino acid that is considered versatile since it is the substrate for the synthesis of many molecules, including NO (Abukhodair et al., 2021). Studies indicate that L-arginine supplementation increases endometrial cell proliferation by a NO-dependent mechanism and increases cell survival during oxidative stress (Suschek et al., 2003; Greene et al., 2013).

The results of treatments with L-NAME increased cell viability in our tests. That can be justified by the fact that NOS isoforms can also generate superoxide anion (Xia et al., 1998; Gonzalez-Vicente et al., 2016; Channon, 2021), an important ROS, which is cytotoxic and capable of affecting the organization of cellular plasma membrane, leading to apoptosis or even necrosis, through the stimulation of inflammatory mediators such as cytokines, oxidized lipoproteins and other types of molecular patterns (Theofilis et al., 2021). Thus, treatment with L-NAME inhibits both NO and superoxide production, and inhibition of superoxide synthesis may contribute to greater cell proliferation, as noted in the results (Kaesemeyer et al., 2000).

Even though ED may reduce cell viability, *in vitro* studies should pay attention to treatments that affect cell survival, since the viability may be affected directly by the drug used in the treatment and not by the pathological condition. In addition, it is known that numerous pathways are involved in cell proliferation and death processes (Anazetti and Melo, 2007). Therefore, for a better study of endothelial dysfunction, we proposed the use of concentrations and time points that least altered cell viability, which were 72 h without L-NAME retreatment and 96 h with L-NAME retreatment.

One of the striking features of endothelial dysfunction is the reduction in NO bioavailability, which can occur either by a reduction in its synthesis or by an increase in its degradation (Vanhoutte et al., 2017; Cyr et al., 2020). A reduction in NO bioavailability could be achieved by treatment with L-NAME because it reduces NO synthesis in a dose-dependent manner, as observed from nitrite quantification results. This is because L-NAME, one of the first synthetic inhibitors of NOS, has good experimental application and is already widespread in investigating NO involvement in different processes (Víteček et al., 2012).

Treatment with L-arginine, most of the time, showed an increase in nitrite concentration, indicating a possible NO synthesis. NO is known to be synthesized from L-arginine as a substrate, and the absence or impairment of L-arginine could reduce the synthesis of NO, characterizing a classical endothelial dysfunction. L-arginine supplementation has been shown to be beneficial for patients with vascular disease, as it contributes to the increase in NO synthesis (Vanhoutte et al., 2017). In addition, this increase in NO synthesis caused by L-arginine indicates good NOS activity in these cells (Arese et al., 1995).

Based on the results of cell viability and indirect NO quantification, it was hypothesized that cells treated with 10µM and 100 μM L-NAME for 72 h without retreatment and for 96 h with retreatment would be able to mimic a condition similar to ED. Retreatment has been used in other assays to ensure that the treatment is available in the cell for its action to be studied, thus reducing the possibility that it will be metabolized and its activity reduced. Here we use this technique to make the method similar to the chronic administration that occurs *in vivo* (Rees et al., 1990; Kopincová et al., 2012).

Endothelial dysfunction is not only characterized by reduced NO bioavailability and another important fact is the increased expression of NOX1, NOX2 and NOX5, associated with inflammation, and reduced production of vasculature-protective biomolecules, such as the product catalyzed by NOX4 (Drummond and Sobey, 2014; Liao et al., 2018; Zhang et al., 2020). Therefore, we investigated the expression of NOX isoform components, p22^phox^ and p47^phox^, already described as expressed in endothelial cells (Jones et al., 1996; Babior, 2000) and as a component of the main isoforms present in these cells, NOX1, NOX2, NOX4 and NOX5 (Drummond and Sobey, 2014). NOX1, NOX2 and NOX5 are characterized by their direct involvement in the onset of inflammation, apoptosis and endothelial dysfunction, while NOX4, by contrast, is characterized as an important vasoprotective agent, involved in the suppression of cell death pathways and increased NO bioviability (Liao et al., 2018; Zhang et al., 2020).

P22^phox^ is an indispensable component of the Nox complex, being indispensable for its activity and stability (Tang et al., 2019). The p47^phox^ in turn is a cytosolic component that acts as an organizer. Our results pointed to a significant reduction of p22^phox^ in cells treated with 100 μM L-NAME for 96 h with retreatment and no amplification of p47^phox^ was observed. That may suggest a higher expression of NOX. Since NOX4 is the most expressed NADPH oxidase in endothelial cells (Drummond and Sobey, 2014), the only isoform that is constitutively expressed and that requires only the p22^phox^ component to be active (Martyn et al., 2006; Nisimoto et al., 2010; Tang et al., 2019), and also in agreement with the results obtained for the analysis of NOX4 expression, our results may indicate a possible reduction in the expression and activity of this enzyme, leading to a diminished NO bioavailability, reinforcing the signals of a condition of endothelial dysfunction (Langbein et al., 2016). Importantly, we must highlight that the expression of p22^phox^ is being demonstrated for the first time in tEnd.1 cells.

Langbein and collaborators (2016) observed in *in vivo* studies that the endothelial function was compromised in the thoracic aorta of mice knockout for the Nox4 gene and in a hyperlipidic diet, causing an endothelial dysfunction (Langbein et al., 2016). NOX isoforms are generally characterized by synthesizing O_2_º−, except for NOX4, which synthesizes H_2_O_2_ (Martyn et al., 2006). While O_2_o− can react with NO and cause the formation of another ROS, peroxynitrite, reducing the bioavailability of NO, H_2_O_2_ does not interact with NO. In addition, research highlights the importance of producing H_2_O_2_, which has a vasodilating action at low concentrations, acting as a hyperpolarizing factor derived from the endothelium (Matoba et al., 2000; Ray et al., 2011; Hu et al., 2017; Morawietz, 2018).

We were also able to visualize the increase in the expression of IL-6 and ACE, mainly in cells treated with L-NAME 100 µM for 96 h with retreatment being a clue of increased pro-inflammatory and vasoconstrictor factors, respectively. About IL-6 it is already known that it is a cytokine that exerts several functions, acting from defense to inflammation. And it can be produced by endothelial cells. This cytokine is produced quickly and contributes to the activation of inflammation in an acute way, stimulating hematopoiesis and immune reactions (Tanaka et al., 2014. Regarding the angiotensin-converting enzyme (ACE), it is known that it acts by converting angiotensin I to angiotensin II, an important vasoconstrictor (Hersh et al., 2022).

Another important feature of this condition is the increased synthesis of ROS, and the consequent oxidative stress (Tian et al., 2017; Incalza et al., 2018). The protocol performed in this study for the treatment with L-NAME did not allow the observation of superoxide production by eNOS, since L-NAME also inhibits superoxide synthesis as mentioned before, being used in experiments aimed at identifying the source of superoxide (Vanhoutte et al., 2009; Varadharaj et al., 2015). In addition to checking gene expression, we sought to visualize another indicative of oxidative stress, which is lipid peroxidation. No significant differences in TBARS test were observed after 72 h without retreatment. Importantly, the absence of lipid peroxidation does not exclude oxidative stress, and other antioxidant enzymes, such as glutathione peroxidase, may have acted, preventing lipid peroxidation (Giblin, 2000).

## Conclusion

**FIGURE 8:**
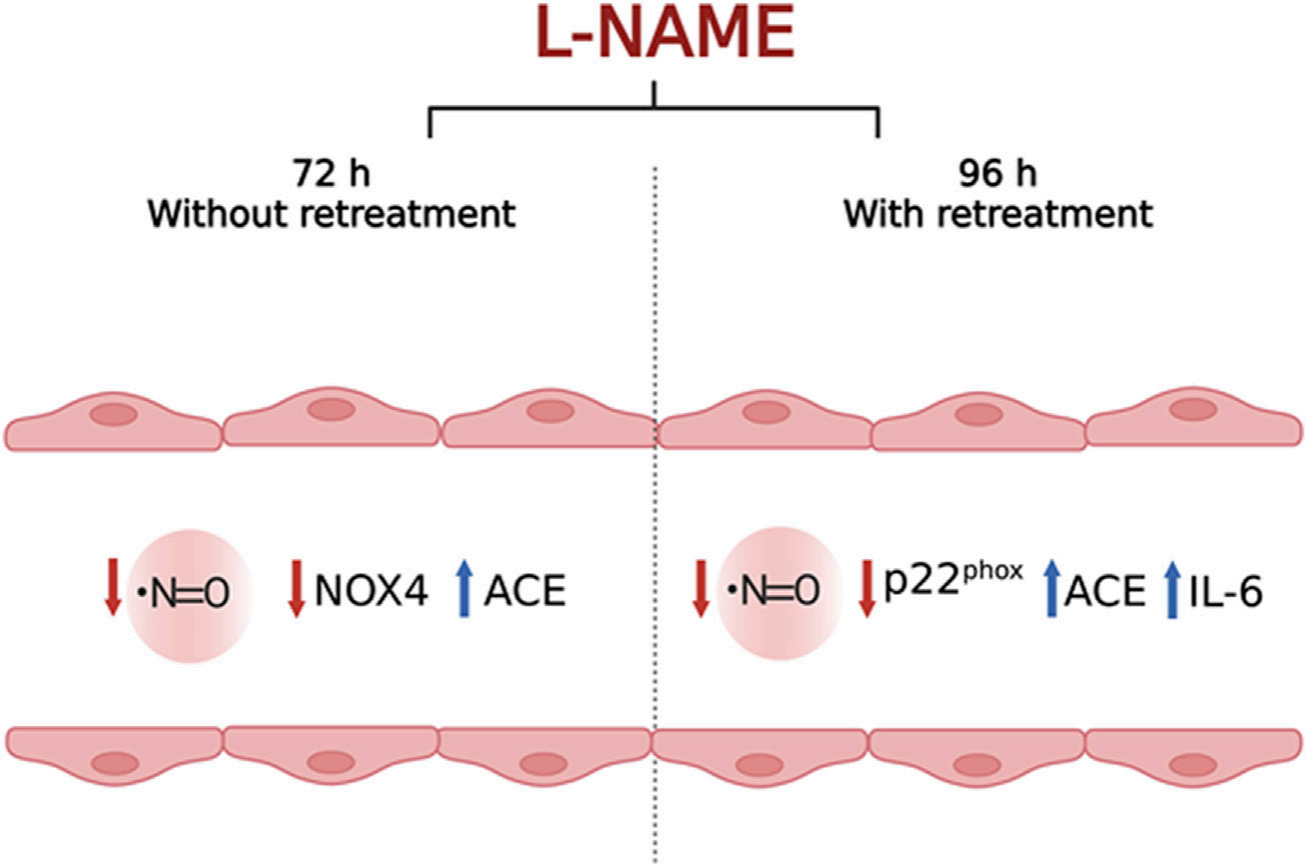
Schematic figure representing the main conclusions of the study. tEnd.1 cells submitted to L-NAME treatment for 72 h without retreatment exhibited decreased NO bioavailability, decreased NOX4 with a possible reduction in vascular protection, and increased ACE transcript levels, possibly associated with increased levels of the vasoconstrictor angiotensin II. At 96 h of L-NAME exposition, endothelial cells showed decreased NO availability, decreased p22^phox^ expression with a possible reduction in NOX4 activity and vascular protection; and increased ACE and IL-6 mRNA levels, indicating increased inflammation and vasoconstriction events, respectively. Both conditions standardized in this study are thus suitable as *in vitro* experimental models to investigate phenomena present in endothelial dysfunction.

Our results indicate that treatment with 100 μM of L-NAME for 72 h without retreatment and 96 h with retreatment were able to differently induce some of the hallmark events of endothelial dysfunction: reduced NO concentration, reduced vascular protection, increased production of vasoconstrictors and IL-6, a pro-inflammatory cytokine. Each of the chosen times had its own characteristics, but they are both associated to ED. Therefore, here we propose that both times can be used as *in vitro* models of ED, but the choice of the best one must be based on the objective of the study. This is because 100 μM L-NAME treatment for 72 h without retreatment reduced NO concentration and NOX4 expression, and increased ACE expression, mimicking reduced vascular protection and increased vasoconstriction. On the other hand, treatment with 100 μM L-NAME for 96 h with retreatment reduced NO concentration and the expression of p22^phox^ gene, while increasing the expression of IL-6 and ACE genes, simulating the increase in inflammation and vasoconstriction processes. Finally, we are proposing a simple, fast, relatively cheap, and feasible protocol to simulate ED based on NO inhibition, using a cellular *in vitro* approach. The conclusions and purpose of this study are depicted in Figure 8.

## Data Availability

The original contributions presented in the study are included in the article/Supplementary Material, further inquiries can be directed to the corresponding author.

## Supplementary material

SUPPLEMENTARY FIGURE S1 Cell viability of the thymus-derived endothelial cell line tEnd.1 after treatment with L-arginine (100 µM) and L-NAME (1, 10, 100 µM, 1 mM) for 12, 24, 48, 72, 96 and 120 h without retreatment. Data (mean ± standard deviation, *n* = 5) were analyzed by Twoway ANOVA, followed by Tukey’s multiple comparisons test. (*represents significant difference between groups, for *p* < 0.0001).

SUPPLEMENTARY FIGURE S2 Cell viability of the thymus-derived endothelial cell line tEnd.1 after treatment with L-arginine (100 µM) and L-NAME (1, 10, 100 µM, 1 mM) for 12, 24, 48, 72, 96 and 120 h with retreatment every 24 h. Data (mean ± standard deviation, *n* = 5) were analyzed by Two-way ANOVA, followed by Tukey’s multiple comparisons test. (*represents significant difference between groups, for *p* < 0.0001).

SUPPLEMENTARY FIGURE S3 Nitrite concentration in culture medium of the thymus-derived endothelial cell line tEnd.1after treatment with L-arginine (100 µM) and L-NAME (1, 10, 100 µM, 1 mM) for 12, 24, 48, 72, 96, and 120 h without retreatment. Data (mean ± standard deviation, *n*=5) were compared by Two-way ANOVA, followed by Tukey’s test. (*represents statistically significant difference compared between groups for *p* < 0.0001).

SUPPLEMENTARY FIGURE S4 Nitrite concentration in culture medium of the thymus-derived endothelial cell line tEnd.1 after treatment with L-arginine (100 µM) and L-NAME (1, 10, 100 µM, 1 mM) for 12, 24, 48, 72, 96 and 120 h with retreatment every 24 h. Data (mean ± standard deviation, *n* = 5) were compared by Two-way ANOVA, followed by Tukey’s test (*represents statistically significant difference compared between groups for *p* < 0.0001).
