# Supplementary figures and images for: Endothelial dysfunction due to the inhibition of the synthesis of nitric oxide: Proposal and characterization of an in vitro cellular model
Source: Front Physiol. 2022 Nov 17;13:978378. doi: 10.3389/fphys.2022.978378 (PMC9714775; doi:10.3389/fphys.2022.978378)

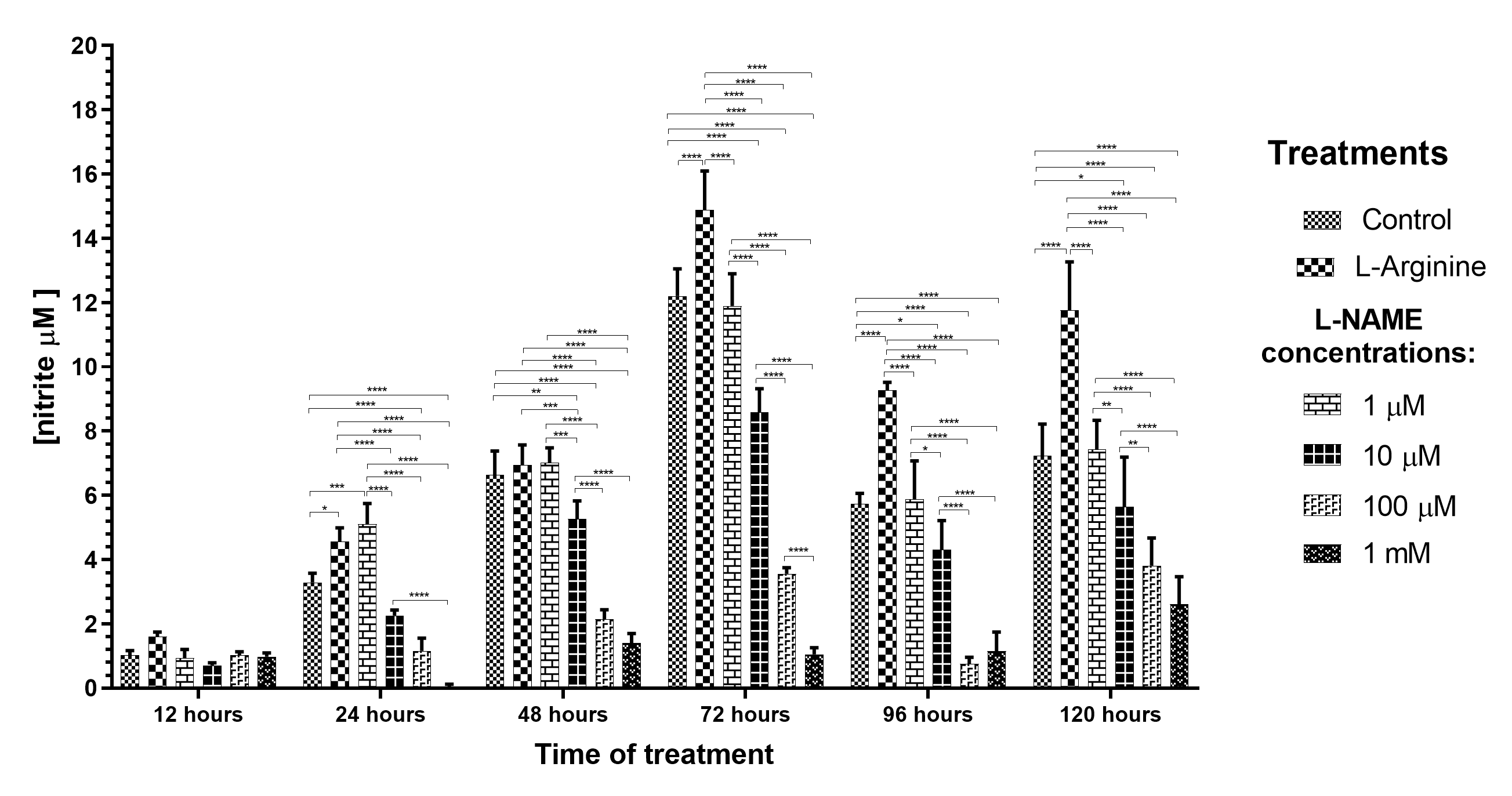

Supplement: Supplementary file 1 [file Image3.TIF]

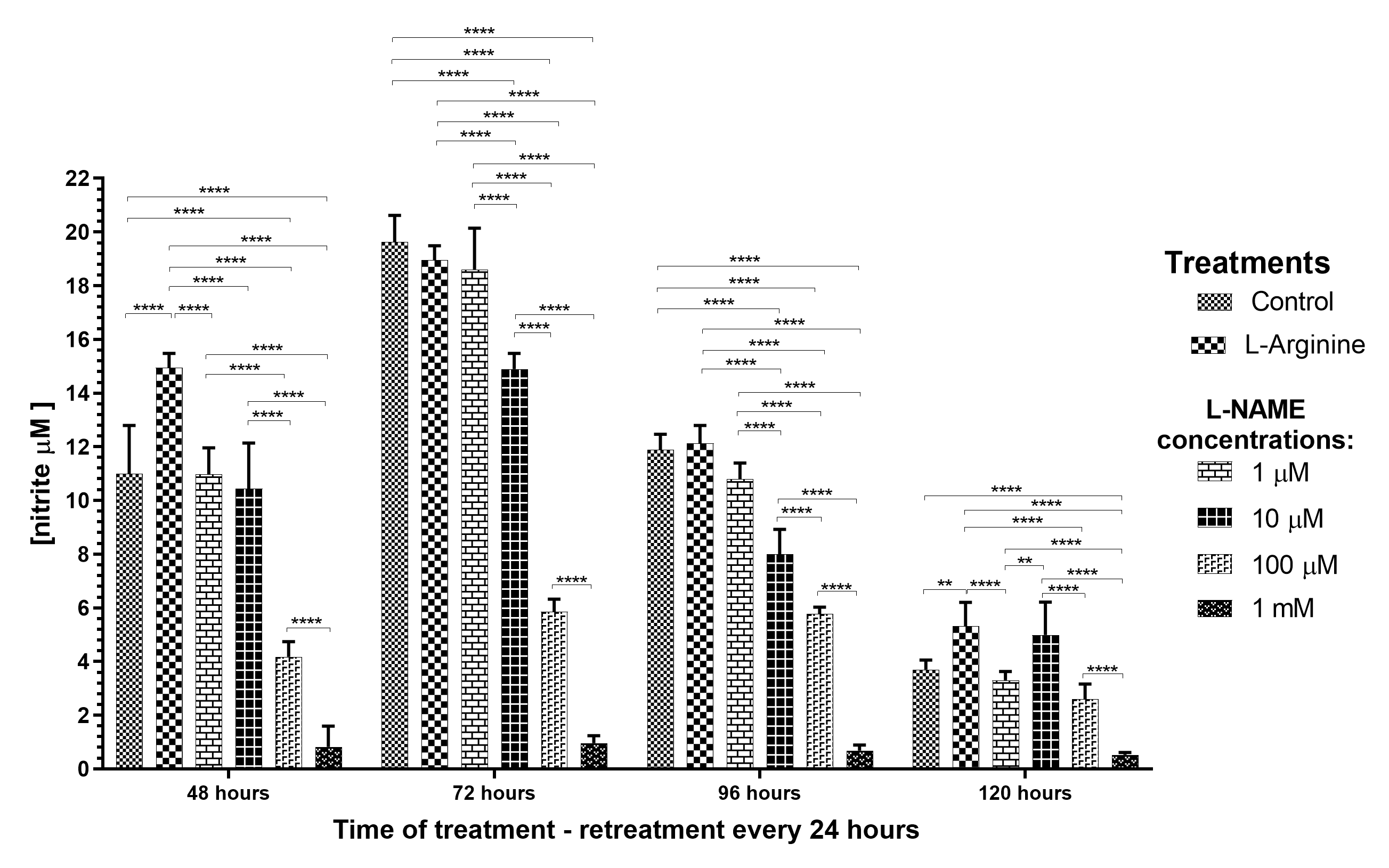

Supplement: Supplementary file 2 [file Image4.TIF]

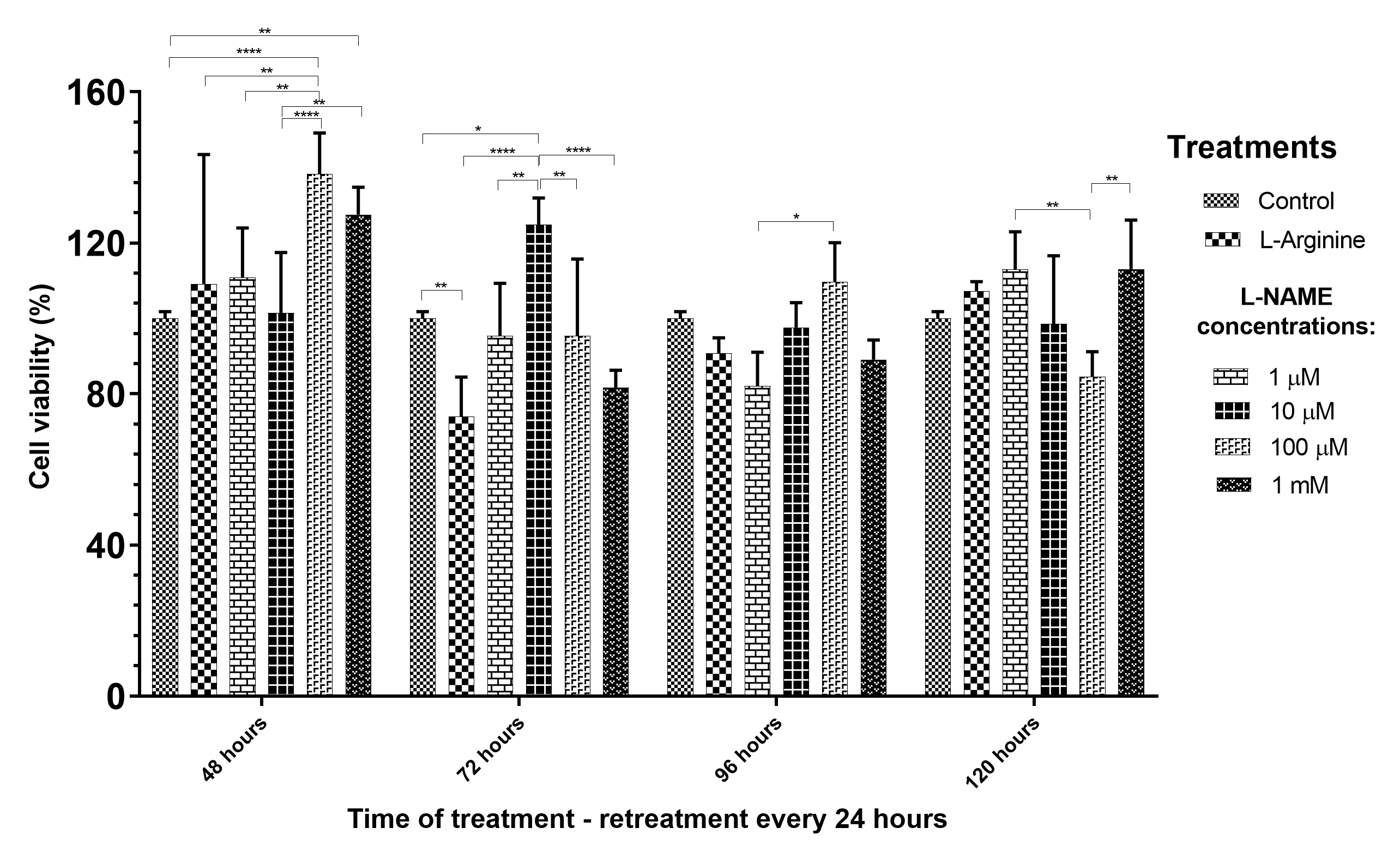

Supplement: Supplementary file 3 [file Image2.TIF]

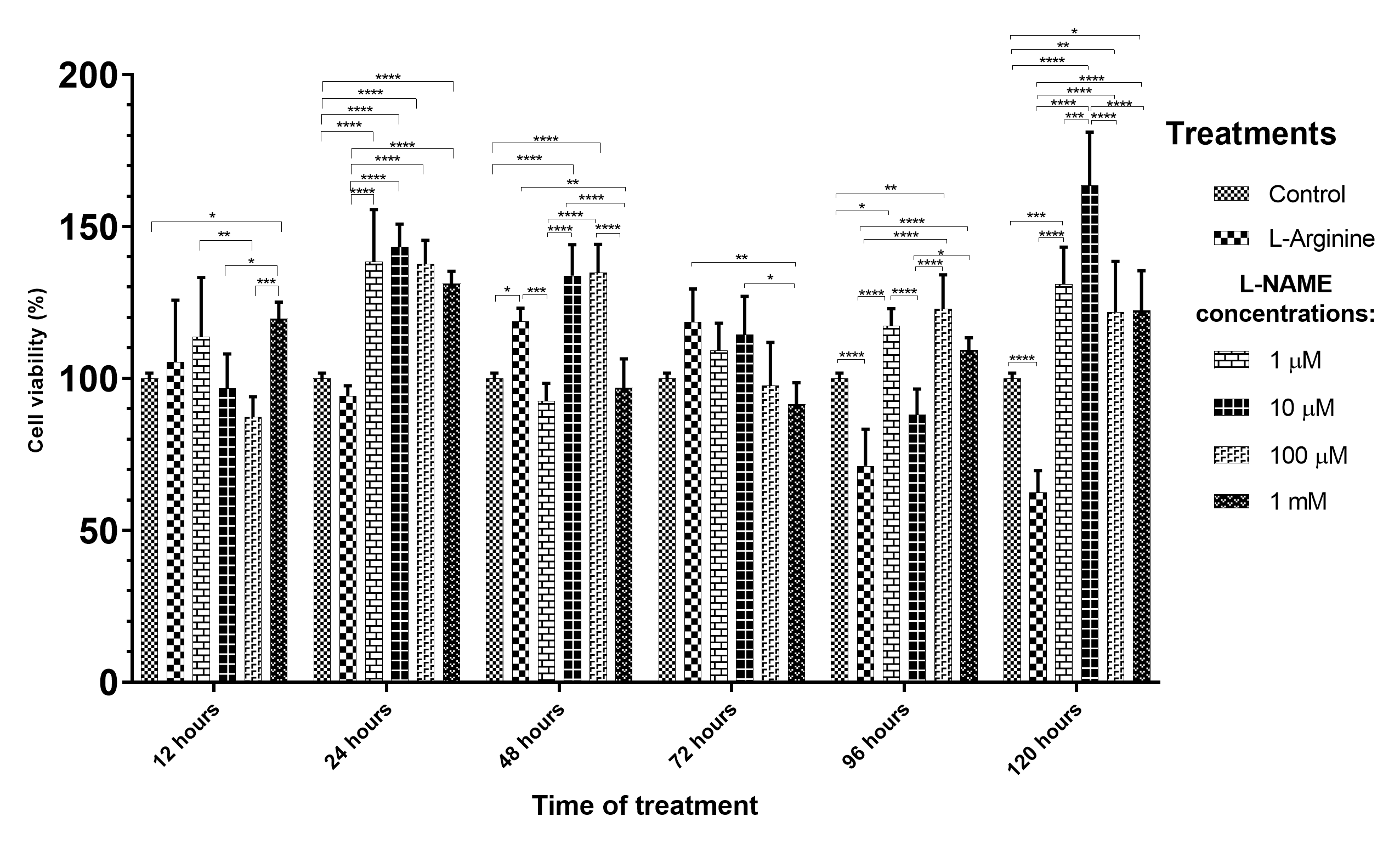

Supplement: Supplementary file 4 [file Image1.TIF]
